# Emergence and clonal expansion of in vitro artemisinin-resistant *Plasmodium falciparum kelch13* R561H mutant parasites in Rwanda

**DOI:** 10.1038/s41591-020-1005-2

**Published:** 2020-08-03

**Authors:** Aline Uwimana, Eric Legrand, Barbara H. Stokes, Jean-Louis Mangala Ndikumana, Marian Warsame, Noella Umulisa, Daniel Ngamije, Tharcisse Munyaneza, Jean-Baptiste Mazarati, Kaendi Munguti, Pascal Campagne, Alexis Criscuolo, Frédéric Ariey, Monique Murindahabi, Pascal Ringwald, David A. Fidock, Aimable Mbituyumuremyi, Didier Menard

**Affiliations:** 1 Malaria and Other Parasitic Diseases Division, Rwanda Biomedical Centre (RBC), Kigali, Rwanda; 2 Malaria Genetics and Resistance Unit–Institut Pasteur, INSERM U1201, CNRS ERL9195, Paris, France; 3 Department of Microbiology and Immunology, Columbia University Irving Medical Center, New York, USA; 4 University of Gothenburg, Gothenburg, Sweden; 5 Maternal and Child Survival Program/JHPIEGO, Baltimore, MD USA; 6 Impact Malaria Rwanda, Kigali, Rwanda; 7 Ministry of Health, Kigali, Rwanda; 8 National Reference Laboratory (NRL), BIOS /Rwanda Biomedical Centre (RBC), Kigali, Rwanda; 9 US President’s Malaria Initiative, Kigali, Rwanda; 10 Hub de Bioinformatique et Biostatistique–Département Biologie Computationnelle, Paris, France; 11 INSERM 1016, Institut Cochin, Service de Parasitologie-Mycologie, Hôpital Cochin, Université de Paris, Paris, France; 12 Roll Back Malaria for West and Central Africa, Kigali, Rwanda; 13 Global Malaria Programme, World Health Organization, Geneva, Switzerland; 14 Division of Infectious Diseases, Department of Medicine, Columbia University Irving Medical Center, New York, NY USA

**Keywords:** Antiparasitic agents, Parasitic infection

## Abstract

Artemisinin resistance (delayed *P. falciparum* clearance following artemisinin-based combination therapy), is widespread across Southeast Asia but to date has not been reported in Africa^1–4^. Here we genotyped the *P. falciparum K13* (*Pfkelch13*) propeller domain, mutations in which can mediate artemisinin resistance^5,6^, in pretreatment samples collected from recent dihydroarteminisin-piperaquine and artemether-lumefantrine efficacy trials in Rwanda^7^. While cure rates were >95% in both treatment arms, the *Pfkelch13* R561H mutation was identified in 19 of 257 (7.4%) patients at Masaka. Phylogenetic analysis revealed the expansion of an indigenous R561H lineage. Gene editing confirmed that this mutation can drive artemisinin resistance in vitro. This study provides evidence for the de novo emergence of *Pfkelch13*-mediated artemisinin resistance in Rwanda, potentially compromising the continued success of antimalarial chemotherapy in Africa.

## Abstract (web summary)

Identification in Rwanda of mutations in *Plasmodium falciparum* capable of conferring in vitro resistance to artemisinin, an essential medicine for the treatment of malaria, underscore the crucial need for surveillance in Africa to safeguard efficacy of life-saving therapies.

## Main

Malaria represents a major public health issue in the tropics, with an estimated 228 million cases and 405,000 deaths in 2018 (refs. ^8,9^). Of increasing concern is *P. falciparum* resistance to artemisinin (ART) derivatives, used worldwide as the core components of ART-based combination therapies (ACTs)^10^. ART resistance (ART-R), characterized by delayed *P.* *falciparum* clearance following treatment with artemisinin monotherapy or an ACT^1,11^, is now widespread in the Greater Mekong subregion (GMS), which consists of Cambodia, Thailand, Vietnam, Myanmar and Laos^12,13^. Resistance to the partner drugs piperaquine and mefloquine is also now common in the GMS, causing high rates of ACT treatment failure^14,15^.

The appearance of ART-R parasites in Africa would pose a major public health threat. Resistance to the former first-line antimalarial chloroquine first arose in the GMS in the 1960s before spreading to Africa. Resistance to pyrimethamine (used in association with sulfadoxine) followed shortly thereafter^16^. The lost clinical efficacy of these compounds is suspected to have contributed to millions of additional malaria deaths in young African children in the 1980s^17^. In addition to the risk of imported resistance^18^, the likelihood of resistance emerging locally in Africa has increased in areas where control measures have reduced the disease transmission intensity. The resulting attenuation in naturally acquired human immunity can increase the frequency of symptomatic infections and the need for treatment, while decreasing parasite genetic diversity and reducing competition between sensitive and resistant parasites^19^. To date, the efficacy of ACTs has remained high outside Southeast Asia (SEA)^2^. Early detection of resistance provides the best chance of minimizing its lethal impact.

Mutations in the *Pfkelch13* propeller domain (PF3D7_1343700) constitute the primary determinant of ART-R^1,5,6^. These mutations are suspected to reduce Pfkelch13 function, which is required for parasite-mediated endocytosis of host hemoglobin in the newly invaded intra-erythrocytic ring stages^20,21^. *Pfkelch13* C580Y is the most widespread allele in SEA^13,15^ and has recently been detected in Guyana^22^ and Papua New Guinea^23^. In Africa, slow-clearing infections after ACT treatment have been observed at frequencies of <1%^24^. Previously we observed nonsynonymous *Pfkelch13* mutations in <5% of African isolates, with >50% of the polymorphisms present in only a single *P.* *falciparum* infection. The most frequent *Pfkelch13* mutation in Africa was A578S, which did not confer ART-R in vivo or in vitro^4^. Nonsynonymous *Pfkelch13* mutations associated with delayed parasite clearance or day 3 positivity (day 3^+^) in the GMS (F446I, Y493H, R539T, I543T, P553L, R561H, P574L, C580Y, A675V) have only been rarely reported, if at all, in African parasites^25,26^.

Here we conducted an in-depth genetic analysis of *P.* *falciparum* samples collected from 2012 to 2015 at six Rwandan sites and performed gene-editing studies to evaluate the in vitro resistance phenotypes of parasites harboring the *Pfkelch13* R561H or P574L mutations identified in these samples.

## Results

### Clinical drug efficacy trial design and outcomes

**Fig. 1:**
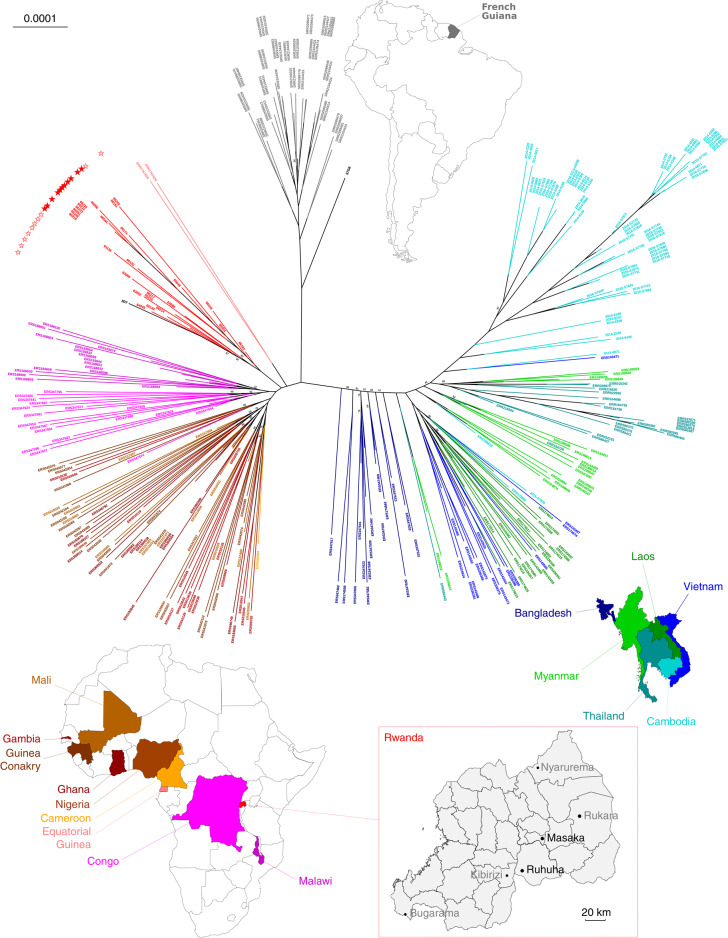
Genome‐wide phylogenetic tree of 25 *P.* *falciparum* Rwandan isolates, together with 315 isolates collected worldwide (Africa, Asia and South America). Isolates were sourced from the MalariaGEN *P.* *falciparum* Community Project (https://www.malariagen.net/apps/pf/4.0). Locations of clinical drug efficacy study sites where Rwandan isolates were collected are indicated. Patients enrolled at Masaka and Ruhuha (black) were treated with AL or DP, whereas patients enrolled at Bugarama, Kibirizi, Nyarurema and Rukara (gray) were treated with AL. *Pfkelch13* nonsynonymous mutations identified in these regions and relative proportions of mutant alleles are detailed in Table 1. Each leaf represents one sample and is colored according to the country of collection. Rwandan parasites carrying the *Pfkelch13* R561H mutation or the *Pfkelch13* WT allele are identified by filled or unfilled red stars at the tip, respectively. Rwandan *Pfkelch13* R561H mutants are closely related to other African samples at a genomic level, demonstrating that they are the product of a local emergence event. Scale bar, 0.0001 nucleotide substitutions per character. Only branch confidence supports <95% are indicated.

From September 2013 to December 2015, clinical drug efficacy studies to assess the efficacy of artemether-lumefantrine (AL) and dihydroartemisinin-piperaquine (DP) for the treatment of uncomplicated *P.* *falciparum* malaria were conducted in patients enrolled at the Masaka and Ruhuha health facilities in Rwanda (Fig. 1; http://www.isrctn.com/ISRCTN63145981). The overall 42-day PCR-corrected efficacies of AL (95.2% (196 of 206); 95% CI, 91.3% to 97.7%) and DP (97.5% (212 of 217); 95% CI, 94.4% to 99.1%) were similar between both sites (*P* = 0.17, log-rank test)^7^. The day 3 positivity rate (day 3^+^), defined as the proportion of patients who were still parasitemic on day 3 after initiation of treatment, was low for both treatments: 1 of 263 (0.4%) for AL and 0 of 264 for DP (Table 1).

**Table 1:** Characteristics of participants and isolates obtained from participants enrolled in clinical drug efficacy studies at Masaka and Ruhuha, 2013–2015, Rwanda

Patients and samples		2013	2014	2015
**Patients (*n*)**		32	102	400
**Site**	Masaka	11	49	208
	Ruhuha	21	53	192
**Antimalarial treatment**				
**AL**		15	49	202
	Masaka	5	23	103
	Ruhuha	10	26	99
**DP**		17	53	198
	Masaka	6	26	105
	Ruhuha	11	27	93
**Parasitemia (per microliter)**				
	Geometric mean	8,922	7,492	8,730
	Median (IQR)	9,478 (3,360–19,656)	7,580 (2,320–19,600)	9,600 (3,200–23,920)
**Day 3**^+^ **rate**^a^		0% (0 of 31)	0% (0 of 102)	0.3% (1 of 394)
**Clinical outcome at day 42**				
Excluded patients^b^				
	AL	6	9	45
	DP	2	7	42
Cured patients				
	AL	9	37	150
	DP	15	45	152
Recrudescent patients				
	AL	0	3	7
	DP	0	1	4
***Pfkelch13*** **genotyping**				
	WT	28	84	354
	Synonymous mutations^c^	1		5
	460 (M > I)	1		
	*469 (C* > *Y)*			*1*
	513 (R > L)			1
	555 (V > A)			2
	**561 (R** > **H)**		**7**	**12**
	*574 (P* > *L)*			*1*
	575 (R > I)	1		1
	578 (A > S)			1
	592 (G > E)/637 (V > I)^d^			1
	605 (E > K)			1
	626 (A > E)			1
	651 (E > K)	1		
	667 (P > R)		2	1

^a^Data were missing for seven patients. ^b^Excluded patients were patients with new infections or those with undetermined or uncertain PCR genotyping data. ^c^Synonymous mutations were G544G (*n* = 1, detected in 2013), T478T (*n* = 2, detected in 2015) and V666V (*n* = 3, detected in 2015). ^d^Polyclonal infection containing two clones with two different *Pfkelch13* nonsynonymous mutations.

The R561H mutation shown in bold font is validated in our report as an ART-R conferring *Pfkelch13* mutation. The C469Y and P574L mutations shown in italic font have been previously associated with delayed clearance following artemisinin monotherapy or ACT treatment.

### *Pfkelch13* genotyping

*Pfkelch13* propeller domain genotyping was performed on 534 pretreatment samples collected at Masaka and Ruhuha. Of the 507 successfully genotyped samples, 35 (6.9%) harbored 1 of 14 different *Pfkelch13* nonsynonymous mutations. These included M460I, C469Y, R513L, V555A, R561H, P574L, R575I, A578S, G592E, E605K, A626E, V637I, E651K and P667R. One sample contained two clones, each with a different mutation (G592E or V637I). The *Pfkelch13* 561H variant, previously associated with delayed parasite clearance following ART monotherapy or ACT treatment in the GMS^26^, was the most predominant mutant. This R561H mutation was observed exclusively in Masaka, where it was present in 7 of 58 samples in 2013–2014 and 12 of 199 samples in 2015 (19 of 257, 7.4%). We also detected two additional *Pfkelch13* mutations (P574L and C469Y) previously associated with delayed parasite clearance^26^. We did not observe significant changes in the proportion of *Pfkelch13* nonsynonymous mutations over time (*P* = 0.3, chi-squared test, 1.92, d.f., 2) at either study site (Table 1).

*Pfkelch13* genotyping was also carried out on 420 additional blood samples collected before AL treatment from patients enrolled in a study following the same clinical protocol that was conducted in 2012–2015 across four sites in Rwanda (ISRCTN63145981; Fig. 1). Among these, ten (2.4%) carried a *Pfkelch13* nonsynonymous mutation (C469F, V487I, V555A, R561H, A578S, A578V or P667R). The *Pfkelch13* R561H mutation was found in a sample from Rukara from a patient who presented a negative Giemsa-stained blood film on day 3 after treatment (day 3^−^) but had recrudescent parasitemia on day 21. A blood sample collected on this day was also found to carry R561H parasites (Supplementary Table 1).

### Relationship between *Pfkelch13* alleles and clinical outcomes

All patients with uncomplicated *P.* *falciparum* infections that had *Pfkelch13* mutant parasites were day 3^−^, with the exception of one patient who was day 3^+^ (80 parasites per µl) and had *P.* *falciparum* parasites harboring the *Pfkelch13* 574L variant (Table 2). Three patients (one in the AL arm and two in the DP arm) presented signs of severe malaria on day 1 and were treated with intravenous artesunate according to national treatment guidelines. These patients were all day 3^+^ and of these, two had *Pfkelch13* mutant infections (either 561H or 626E).

**Table 2:** Parasitemia at day 3 and PCR-corrected clinical outcome at day 42 of the patients enrolled in clinical drug efficacy studies in Masaka and Ruhuha (2013–2015, AL or DP) and Bugarama, Kibirizi, Nyarurema and Rukara (2012–2015, AL), according to *Pfkelch13* genotypes detected in *P.* *falciparum* isolates collected before ACT treatment

Clinical data	*Pfkelch13* genotype	
	WT/synonymous mutant	Nonsynonymous mutant
**Masaka and Ruhuha 2013–2015**		
Parasitemia at day 3		
Positive	0	1^a^ (AL)
Negative	469 (AL = 237, DP = 232)	33^b^ (AL = 16, DP = 17)
Clinical outcome at day 42		
Cured	373 (AL = 185, DP = 188)	21 (AL = 7, DP = 14)
Recrudescent	9 (AL = 5, DP = 4)	0
**Bugarama, Kibirizi, Nyarurema and Rukara 2012–2015**		
Parasitemia at day 3		
Positive	2	0
Negative	403	10^c^
Clinical outcome at day 42		
Cured	312	7^d^
Recrudescent	14	1^e^

^a^574L. ^b^469Y, 513L, 578S, 592E + 637I, 605K, 651K, 555A, 575I (*n* = 2), 667R (*n* = 3), **561H** (*n* = 18).

^c^578S, 578V, 469F, 667R, **561H**, 487I, 555A (*n* = 4); ^d^578S, 578V, 469F, 667R, 555A (*n* = 3). ^**e**^**561H**. For all mutations *n* = 1 unless otherwise indicated.

By excluding the intravenous artesunate-treated patients presenting with severe malaria from our final analysis, we did not find any association between *Pfkelch13* nonsynonymous mutations and delayed parasite clearance as assessed by day 3^+^ (*P* = 0.06, Fisher’s exact test) or by clinical outcome at day 42 (cured versus recrudescent) (*P* = 1, Fisher’s exact test; Table 2). Furthermore, we did not observe any correlation between mutation status and clinical outcome in the samples from the second study conducted at the four additional sites in Rwanda (*P* = 1 for day 3^+^ and *P* = 0.3 for day 42 clinical outcome, Fisher’s exact test). This analysis included the patient mentioned above (treated with AL) who had a recrudescent parasitemia on day 21 with a *Pfkelch13* 561H infection (Table 2).

### In vitro susceptibility of *Pfkelch13* 561H and 574L mutants to artemisinin

**Fig. 2:**
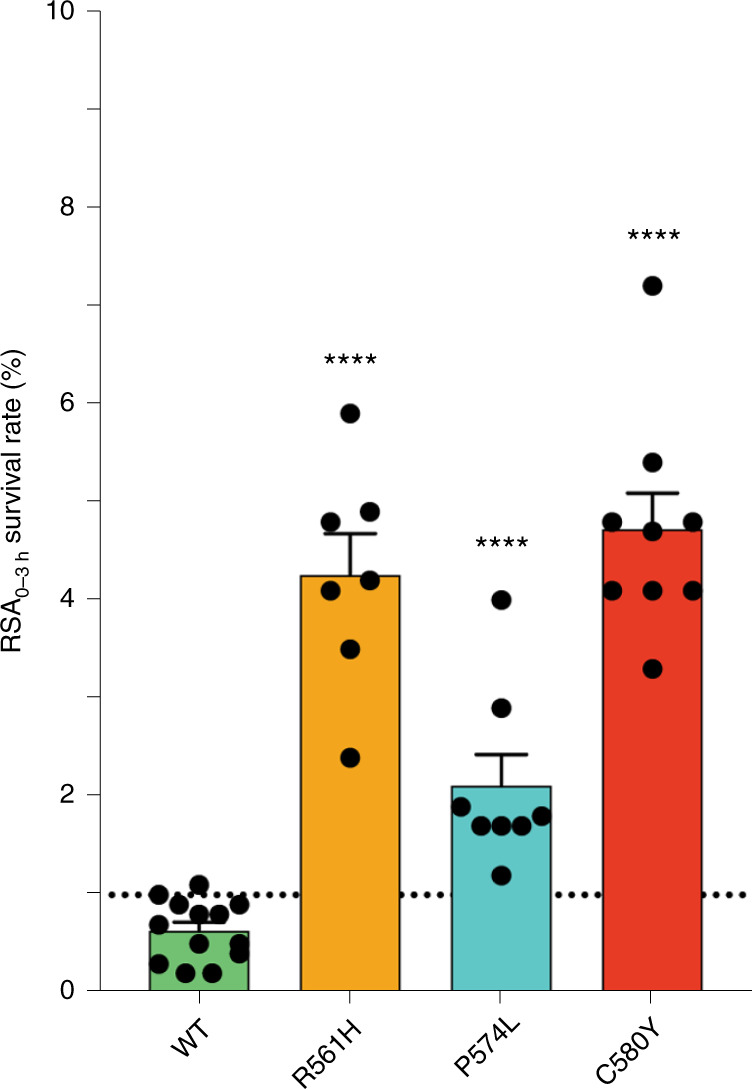
Survival rates of Dd2^R561H^, Dd2^P574L^, Dd2^C580Y^ and Dd2^WT^ lines in the ring-stage survival assay (RSA_0–3h_). Mean ± s.e.m. RSA_0–3h_ survival rates (percentage of viable parasites) were as follows: Dd2^R561H^ 4.3 ± 0.1% (*n* = 7 assays); Dd2^P574L^ 2.1 ± 0.3% (*n* = 8 assays); Dd2^C580Y^ 4.7 ± 0.4% (*n* = 9 assays); Dd2^WT^ 0.6 ± 0.1% (*n* = 13 assays). All assays were performed in duplicate. Mann–Whitney *U*-tests (two-sided) were used to test for statistically significant differences between *Pfkelch13*-edited clones and the Dd2^WT^ comparator line. Survival rates of Dd2^R561H^, Dd2^P574L^ and Dd2^C580Y^ all differed significantly from Dd2^WT^ (**** P < 0.0001). The limit of detection of viable parasites was estimated at 0.1% parasitemia (lower limit of 50 parasitized red blood cells per total number of 50,000 counted for each line in each assay).

To test the impact of the *Pfkelch13* R561H and P574L mutations on ART-R in vitro, we used CRISPR-Cas9 to introduce these mutations into Dd2 parasites and subjected the recombinant mutant and wild-type (WT) control lines to phenotyping in the ring-stage survival assay (RSA_0–3h_)^6^. The *Pfkelch13* R561H mutation was found to confer in vitro ART-R (increased RSA survival), with Dd2^R561H^ parasites exhibiting a mean survival rate of 4.3% versus 0.6% for the Dd2^WT^ line expressing WT *Pfkelch13* (*P* < 0.0001, Mann–Whitney *U*-test). The survival of the 561H line was comparable to that of Dd2^C580Y^ line (mean survival of 4.7%), which harbors the *Pfkelch13* C580Y mutation (Fig. 2). These results demonstrate that the *Pfkelch13* R561H mutation can yield ring-stage ART-R at a level that is comparable to the C580Y mutation that has swept across SEA^13,15^.

Dd2^P574L^ parasites displayed a mean RSA survival rate of 2.1%, which represented a modest but significant increase relative to the Dd2^WT^ line (*P* < 0.0001, Mann–Whitney *U*-test; Fig. 2). These results provide evidence that the *Pfkelch13* P574L mutation is able to confer a lesser degree of ART-R.

### Origins of the Rwandan *Pfkelch13* 561H haplotype and its relationship to other *P.falciparum* populations

To study the origin of the *Pfkelch13* 561H mutants found in Rwanda, we compared whole-genome sequences of 340 samples, comprising 25 Rwandan *P.* *falciparum* sequences generated for this study, and an earlier collection of 104 sequences from central, western and southern African locations, 164 from Bangladesh and SEA and 45 from South America, in addition to 2 reference genomes (3D7 from Africa and 7G8 from South America; Supplementary Table 2). Of the 25 Rwandan sequences, 16 were *Pfkelch13* 561H mutants and 9 were *Pfkelch13* WT. The isolates from SEA (Myanmar and Thailand) included 17 561H mutants. All other parasite sequences either had distinct nonsynonymous *Pfkelch13* mutations or were WT for *Pfkelch13*.

A maximum-likelihood phylogenetic tree inferred from the 14 *P.* *falciparum* chromosomes showed clear separations between the African, Asian and South American parasites (Fig. 1). Additionally, the Rwandan *Pfkelch13* 561H mutants clustered unambiguously with the Rwandan *Pfkelch13* WT parasites.

**Extended Data Fig. 1:**
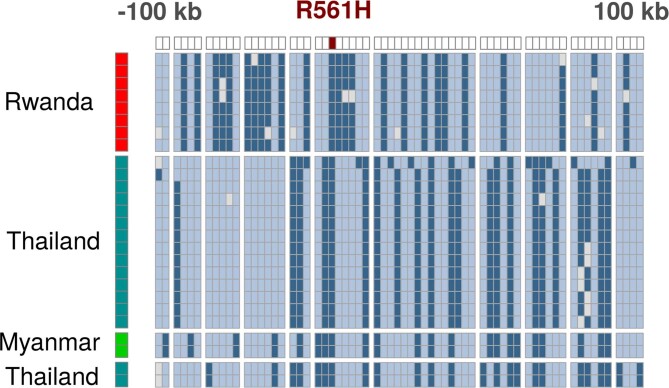
Comparison of mutant pseudo-haplotypes in a 200 kb window around the R561H mutation (100 kb on both sides of the mutation, on chromosome 13). Each cell represents a single SNP. The blocks of cells (grouped in columns) correspond to SNPs falling into the same 20 kb interval within the 200 kb window. The R561H mutation in *Pfkelch13* (PF3D7_1343700) is flagged in the dark red cell at the top. Light blue cells correspond to the reference allele (that is the 3D7 genome), dark blue cells correspond to the alternate allele and grey cells to missing values. Each row corresponds to one isolate, with isolates color-coded according to the country of origin (red for Rwanda, cyan for Thailand and green for Myanmar). Mutant pseudo-haplotypes include eight *P. falciparum* monoclonal Rwandan samples and 18 Southeast Asian samples (from Myanmar and Thailand, sourced from the *Plasmodium falciparum* Community Project; https://www.malariagen.net/apps/pf/4.0). The presence of a single shared haplotype surrounding the R561H mutation in Rwandan *P. falciparum* isolates is consistent with a single epidemiological origin of the genetic background on which the mutation arose. This genetic background demonstrates no genetic relatedness to R561H mutants previously detected in Myanmar and Thailand.

We also explored haplotype diversity across a 200-kb region surrounding the R561H mutation. This analysis used sequences from eight Rwandan *Pfkelch13* mutant infections that seemed to be predominantly monoclonal (allelic depth of the WT allele <0.05), as well as 17 sequences from *Pfkelch13* 561H mutants from SEA (Myanmar and Thailand). The presence of a single shared haplotype surrounding the 561H variant in the Rwandan samples was consistent with a single epidemiological origin for this mutation. These results confirmed that the Rwandan 561H mutants share no genetic relatedness to the 561H mutants previously detected in Myanmar and Thailand (Extended Data Fig. 1).

**Extended Data Fig. 2:**
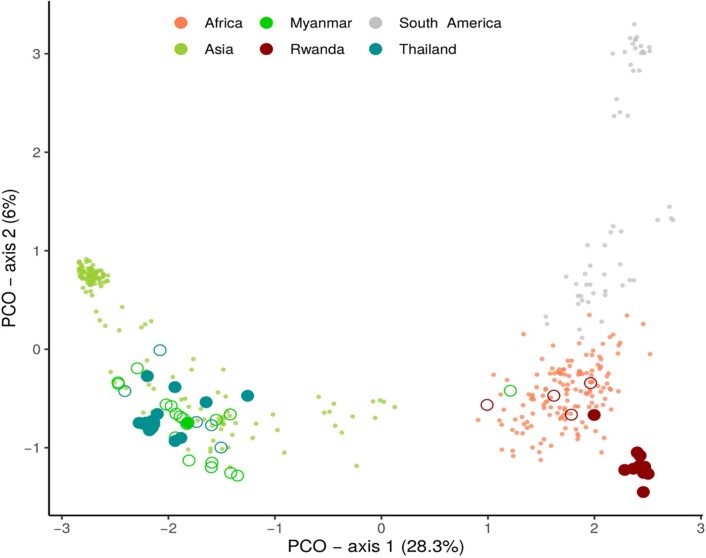
Principal Coordinate Analysis (PCoA) based on pairwise genetic distances in a 494 kb window around the *Pfkelch13* gene. Principal Coordinate Analysis including *Pfkelch13* wild type and 561H isolates including those sourced from a public database (small dots, the MalariaGEN *Plasmodium falciparum* Community Project, https://www.malariagen.net/apps/pf/4.0) and originating from different continents (Asia, Africa or South America). Isolates originating from populations where the *Pfkelch13* R561H mutation was found are emphasized (large dots). Empty large dots correspond to *Pfkelch13* wild-type isolates and filled large dots correspond to *Pfkelch13* 561H mutants. While the mutants tend to cluster with individuals of similar origin, axis 1 clearly discriminates African (Rwanda) from Asian (Thailand and Myanmar) *Pfkelch13* 561H mutants.

**Extended Data Fig. 3:**
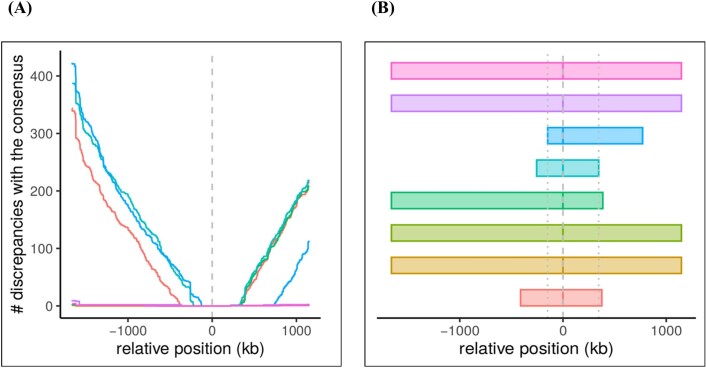
Extent of the common core haplotype in the eight Rwandan *Pfkelch13* 561H isolates (monoclonal isolates). **a**, Recombination breakpoints estimated based on the accumulation of discrepancies between the consensus core sequence of mutants and each haplotype on both sides of the *Pfkelch13* R561H mutation. The analysis was performed on the eight isolates that appeared monoclonal. Genomic positions are indicated relative to the *Pfkelch13* mutation (0 kb). **b**, Length of the corresponding core mutant haplotypes (obtained based on (A)). Dotted lines delineate a common core region of 494 kb within which all mutant haplotypes appear identical. Genomic positions are indicated relative to the *Pfkelch13* mutation (relative position 0 kb). In the larger haplotypes, no clear recombination breakpoint was observed on chromosome 13, indicating a sequence identity along the whole chromosome. Each of the eight isolates are represented by a specific color, consistent between panel (**a**) and panel (**b**).

Next, we performed a principal coordinate analysis (PCoA) based on a pairwise genetic distance matrix (computed from a 200-kb window around the *Pfkelch13* gene). This analysis confirmed that the African samples (including both the Rwandan *Pfkelch13* 561H mutants and WT parasites) clustered together and were distinct from Asian samples (Extended Data Fig. 2). In the eight *Pfkelch13* 561H mutants from Rwanda we identified an extended 494-kb region, encompassing the mutation that was identical across isolates (Extended Data Fig. 3). Although an ancient common ancestry cannot strictly be ruled out, our data provide compelling evidence that Rwandan *Pfkelch13* 561H is the product of a recent de novo local emergence.

### Investigation of the genetic background of Rwandan *Pfkelch13* 561H mutants

To investigate further the genetic background of the Rwandan *Pfkelch13* 561H mutants, we searched for molecular signatures associated with resistance to other antimalarials, including the ACT partner drugs piperaquine and lumefantrine. We also screened for mutations that have been identified in founder populations common to SEA ART-R parasites (those that constitute a ‘genetic background’ for ART-R)^27^.

First, we investigated 233 isolates from Masaka and Ruhuha for amplification of the plasmepsin2 (*pfpm2;* PF3D7_1408000) and multidrug resistance-1 (*pfmdr1;* PF3D7_0523000) genes, considered markers of reduced susceptibility to piperaquine and lumefantrine/mefloquine, respectively^28–30^. Of these, we found 4 isolates (1.7%) with two copies of *pfpm2* and 12 isolates (5.1%) with two copies of *pfmdr1*. All isolates carrying two copies of *pfpm2* or *pfmdr1* were WT for *Pfkelch13* (Supplementary Table 3). We also tested 14 of the 20 *Pfkelch13* 561H mutants for mutations in the chloroquine resistance transporter gene (*pfcrt*; PF3D7_0709000), whose variants can confer resistance to chloroquine or piperaquine^31,32^. All 561H mutants carried WT *pfcrt* (Supplementary Table 4).

Second, we tested whether the proportions of single-nucleotide polymorphisms (SNPs) associated with the emergence of ART-R in the SEA genetic background varied between Rwandan *Pfkelch13* 561H mutants and WT parasites. For this analysis, we used 14 Rwandan *Pfkelch13* 561H mutants and 10 randomly selected WT parasites and tested for mutations in the six markers defining the SEA ART-R background. No significant differences were observed between the two groups of isolates. We detected four isolates with the D193Y mutation in the ferredoxin gene (*pffd*; PF3D7_1318100), two (15.4%) in *Pfkelch13* 561H mutant samples and two (22.2%) in WT samples (*P* = 0.69, Fisher’s exact test). No mutations were detected in the *P.* *falciparum* apicoplast ribosomal protein S10 precursor *(pfarps10*, PF3D7_1460900*)*, multidrug resistance protein 2 *(pfmdr2*, PF3D7_1447900*), pfpib7 (*PF3D7_0720700), *pfpph* (PF3D7_1012700) or exonuclease (PF3D7_1362500) genes in either the *Pfkelch13* 561H or WT isolates (Supplementary Table 4).

## Discussion

This study clearly shows early warning signs of ART-R in Rwanda. We provide evidence for the clonal expansion of an indigenous *Pfkelch13* 561H lineage in two localities 100 km apart in Rwanda (prevalence 7.4% in Masaka and 0.7% in Rukara). This expansion was not linked to delayed parasite clearance in vivo or clinical treatment failure following AL or DP treatments, likely due to the high efficacy of the partner drugs lumefantrine and piperaquine. Genetic analyses indicate that Rwandan *Pfkelch13* 561H mutants are the product of recent de novo local emergence. These findings contrast with previous scenarios from the 1980s in which the emergence of chloroquine- and pyrimethamine-resistant parasites in Africa resulted from the westward spread of these parasites from SEA^16^, and confirm that local emergence of ART-R is possible in Africa.

We used gene editing and the RSA_0–3h_, a clinically validated in vitro phenotypic analysis^6,33^, to demonstrate that the *Pfkelch13* R561H mutation is sufficient to confer ART-R in vitro. These experiments employed Dd2, which has been the most widely used *P.* *falciparum* strain for *Pfkelch13* gene editing^4,6^. Our results revealed that in Dd2 parasites, the R561H mutation confers survival at levels comparable to the C580Y mutation that predominates in SEA (with mean survival rates of 4.3% and 4.7%, respectively)^1,13,15^. Previous studies have shown that *Pfkelch13* mutations that afford resistance do so across all strains, with the parasite genetic background modulating resistance levels and with mutations conferring less resistance in Dd2 compared to contemporary SEA strains^6^. While we did not test the impact of this mutation in Rwandan parasites due to a lack of availability of culture-adapted strains, we are confident that the resistance phenotype observed herein would be maintained across strains.

At a genomic level, Rwandan *Pfkelch13* 561H mutants were phylogenetically closely related to other African samples and clustered unambiguously with Rwandan *Pfkelch13* WT parasites. Haplotype analysis revealed that Rwandan *Pfkelch13* 561H mutants shared an identical haplotype surrounding the R561H mutation that differed from the haplotypes of SEA 561H mutants, strongly suggesting a single de novo epidemiological origin and recent spread of the mutation. No genetic relatedness was observed between Rwandan *Pfkelch13* 561H parasites and *Pfkelch13* 561H mutants previously detected in Myanmar and Thailand by PCoA.

The current rise and expansion of the in vitro ART-R *Pfkelch13* R561H mutation in Rwanda is particularly notable in light of the observed absence of clinical outcomes typically associated with ART-R. We suspect that the absence of delayed parasite clearance in Rwandan patients harboring *Pfkelch13* 561H mutant parasites is due to high levels of naturally acquired immunity to *P.* *falciparum* in the study participants. Indeed, it has been shown that *P.* *falciparum* antibody titers are strongly associated with faster parasite clearance rates in patients living in high-transmission areas like Rwanda and that antibodies against *P.* *falciparum* blood stages enhance antimalarial efficacy^34^. In our study, the ages of patients enrolled at both sites ranged from 1 to 14 years, with an estimated median age of 8 years (interquartile range (IQR): 5–11 years). Given that immunity is acquired gradually with age, a clinical drug efficacy trial limited to younger populations (≤5 years of age) might reveal a significant association between the presence of *Pfkelch13* 561H mutants in pretreatment isolates and delayed parasite clearance. We hypothesize that early signs of clinical ART-R can lie undetected in populations with high levels of immunity, calling into question the relevance of the current clinical metrics used to detect ART-R in Africa. This hypothesis is supported by data from population-based mathematical modeling^19^ that showed that ART-R parasites might be able to circulate up to 10 years longer without detection in high-transmission areas than in low-transmission areas.

To date, the *Pfkelch13* R561H mutation has been reported multiple times in SEA (Cambodia until 2006, Myanmar and Thailand)^25^, once in India^35^ and a few times in Africa (Democratic Republic of the Congo^4^, Rwanda^36^ and Tanzania^37^), but has only been associated with slow-clearing infections in SEA^26^. Thus, the degree to which *Pfkelch13* 561H mutant parasites are able to withstand exposure to ART in vivo and how *Pfkelch13* 561H is successfully transmitted between patients in the absence of clinical recrudescence (Table 2) requires further elucidation. It is possible that the resistance advantage afforded by the *Pfkelch13* 561H mutation is slight and undetectable based on day 3^+^ and recrudescence metrics, and thus would be evident only with ART monotherapy trials. Regarding transmission, we can offer several hypotheses. First, *Pfkelch13* 561H mutants could be less susceptible to ART due to an ability to enter into a dormant state^38^ and later produce transmissible gametocytes. Second, *Pfkelch13* 561H parasites may have a higher capacity to be transmitted due to an unknown genetic feature or *Pfkelch13* 561H gametocytes may be less susceptible to the gametocytocidal activity of artemisinin. However, it is most likely that the transmission of *Pfkelch13* 561H mutants in Rwanda is maintained by asymptomatic individuals or mildly symptomatic untreated patients with circulating *Pfkelch13* 561H mutants that have been selected by low levels of circulating drugs.

We did not detect the combination of background mutations earlier suspected to be linked to the ART-R phenotype in SEA in the *Pfkelch13* 561H Rwandan isolates^27^. This suggests that the emergence of mutant *Pfkelch13* that drives in vitro resistance is not dependent on the presence of secondary mutations within the parasite genome. So far, no gene-editing and in vitro phenotyping experiments have been performed to test the importance of these secondary mutations for resistance. Data from this study suggest that mutations in *fd*, *mdr2*, *arps10* and others represent the genetic architecture of regional ART-R in *P.* *falciparum* SEA parasite populations rather than secondary determinants of resistance.

The findings of this study have substantial implications for public health in confirming the de novo emergence and clonal expansion of an ART-R *Pfkelch13* R561H lineage in Rwanda and in validating this mutation as a mediator of ART-R in vitro. In the absence of effective strategies to contain the spread of resistance across Rwanda and to neighboring countries, we may soon witness a rise of resistance to ACT partner drugs, which will in turn lead to high treatment failure rates, as has occurred in SEA^14^. Recent studies have predicted that ACT treatment failures in Africa could be responsible for an additional 78 million cases and 116,000 deaths over a 5-year period^39^.

Molecular surveillance of *Pfkelch13*-related ART-R currently implemented by the National Malaria Control Programme in Rwanda needs to be sustained and strengthened so that mutations can be identified before clinical phenotypes become apparent. Our findings argue for the need for more rapid collection of data, analysis and dissemination of information using new high-throughput field-based surveillance tools operable at a national level. Likewise, we have to reappraise the performances of the current clinical phenotypic metrics (delayed parasite clearance and day 3^+^) to detect the warning signs of ART-R in African populations with high immunity early on.

## Methods

### Clinical drug efficacy trial oversight and blood sample collection

The clinical drug efficacy trial (ISRCTN63145981, http://www.isrctn.com/ISRCTN63145981) was conducted by the Rwanda National Malaria Program between 2013 and 2015 at two health facilities in Rwanda (Masaka and Ruhuha, in the Kicukiro and Bugesera districts, respectively) to assess the efficacy of AL or DP for the treatment of uncomplicated *P*. *falciparum* malaria in children 1–14 years of age, presenting with suspected uncomplicated *P*. *falciparum* malaria^7^. Patients at both sites were randomly assigned to receive a full course of AL (Co-artem, 20 mg artemether and 120 mg lumefantrine per tablet) or DP (Duo-cotecxin, 40 mg dihydroartemisinin and 320 mg piperaquine per tablet) according to the manufacturer’s dosing schedule.

The primary outcome of the study was the PCR-adjusted clinical response to the designated treatment on day 42 (ref. ^40^). The secondary outcome was the day 3^+^, defined as the proportion of patients who were still parasitemic on day 3 after initiation of treatment as assessed by thick blood smear (Supplementary Methods 1)^41^.

### *Pfkelch13* genotyping and whole-genome sequencing

Genomic investigations were carried out on blood samples collected before ACT treatment (AL or DP) from patients enrolled at Masaka and Ruhuha. We also analyzed blood samples collected before AL treatment from patients enrolled in clinical drug efficacy studies conducted at four additional sites (Bugarama, Kibirizi, Nyarurema and Rukara) across Rwanda between 2012 and 2015.

Parasite DNA was extracted from dried blood samples (Fig. 1) using the QIAamp DNA Blood Mini Kit (Qiagen). Mutations in the propeller domain of *Pfkelch13* (PF3D7_1343700, codons 440–680, 720 bp) were identified by capillary sequencing of PCR products^4^. Parasite whole-genome sequences were obtained by Illumina paired-end sequencing after capture-based enrichment of parasite DNA (Supplementary Methods 2)^5^.

### Phylogenetic analysis

For each sequenced sample, read alignments against the chromosome sequences of *P.* *falciparum* 3D7 v45 were processed to infer consensus sequences. These consensus sequences were pooled and concatenated, leading to 17,313,072 aligned nucleotide characters that were used to infer a maximum-likelihood phylogenetic tree (Supplementary Methods 3).

### Genotyping and haplotype analysis

The Genome Analysis Toolkit Haplotype Caller was used to identify SNPs in each isolate. We assessed the genetic identity of *Pfkelch13* 561H mutants from Rwanda and Asia by comparing alleles at loci within a 200-kb window around the mutation and recording the number of discrepancies between each sample and the mutant consensus sequence. PCoA was performed by computing pairwise Euclidean genetic distances between samples in an extended 494-kb window (Supplementary Methods 4).

### Generation of gene-edited lines and in vitro susceptibility assays

Dd2^R561H^ and Dd2^P574L^ gene-edited parasite lines, as well as Dd2^WT^ and the Dd2^C580Y^ lines used as controls, were generated by CRISPR-Cas9-mediated editing of the *Pfkelch13* locus using the pDC2-cam-coSpCas9-U6-hdhfr vector. In vitro ART susceptibilities of these lines were assessed using RSA_0–3h_ (Supplementary Methods 5–7).

### Statistical analysis

Sample size calculations and clinical data management methods have been previously described^7^. PCR-adjusted clinical efficacy rates at day 42 were calculated using Kaplan–Meier survival analysis. Survival curves were compared using the Mantel–Haenszel log-rank test (one-sided). Patients with new infections during the 42-day follow-up period and patients with undetermined or noninterpretable PCR genotyping data were excluded from the final analysis. Data were reported in Microsoft Excel (Office 2016) and analyzed with MedCalc v.12 (MedCalc Software) and Prism 8 (GraphPad Software). Mann–Whitney *U-*tests (two-sided) were used for nonparametric comparisons. For frequency data (expressed with percentages and 95% CIs), we used chi-squared or Fisher’s exact tests (one-sided). Relative risks were estimated using the Mantel–Haenszel test. All *P* values <0.05 were deemed significant.

### Reporting Summary

Further information on research design is available in the Nature Research Reporting Summary linked to this article.

## Online content

Any methods, additional references, Nature Research reporting summaries, source data, extended data, supplementary information, acknowledgements, peer review information; details of author contributions and competing interests; and statements of data and code availability are available at 10.1038/s41591-020-1005-2.

## Supplementary information

Supplementary Information Supplementary Methods 1–7 and Supplementary Tables 1–4.

Reporting Summary

## Data Availability

The data that support the findings of this study are available from the corresponding authors upon request. Parasite whole-genome sequences have been deposited in the repository https://www.ncbi.nlm.nih.gov/bioproject/PRJEB38946, and the sequence files are accessible under the accession numbers ERS4758427 to ERS4758451.
