# Author Correction: Emergence and clonal expansion of in vitro artemisinin-resistant *Plasmodium falciparum kelch13* R561H mutant parasites in Rwanda

**DOI:** 10.1038/s41591-021-01365-y

**Published:** 2021-05-27

**Authors:** Aline Uwimana, Eric Legrand, Barbara H. Stokes, Jean-Louis Mangala Ndikumana, Marian Warsame, Noella Umulisa, Daniel Ngamije, Tharcisse Munyaneza, Jean-Baptiste Mazarati, Kaendi Munguti, Pascal Campagne, Alexis Criscuolo, Frédéric Ariey, Monique Murindahabi, Pascal Ringwald, David A. Fidock, Aimable Mbituyumuremyi, Didier Menard

**Affiliations:** 1 Malaria and Other Parasitic Diseases Division, Rwanda Biomedical Centre (RBC), Kigali, Rwanda; 2 Malaria Genetics and Resistance Unit–Institut Pasteur, INSERM U1201, CNRS ERL9195, Paris, France; 3 Department of Microbiology and Immunology, Columbia University Irving Medical Center, New York, USA; 4 University of Gothenburg, Gothenburg, Sweden; 5 Maternal and Child Survival Program/JHPIEGO, Baltimore, MD USA; 6 Impact Malaria Rwanda, Kigali, Rwanda; 7 Ministry of Health, Kigali, Rwanda; 8 National Reference Laboratory (NRL), BIOS /Rwanda Biomedical Centre (RBC), Kigali, Rwanda; 9 US President’s Malaria Initiative, Kigali, Rwanda; 10 Hub de Bioinformatique et Biostatistique–Département Biologie Computationnelle, Paris, France; 11 INSERM 1016, Institut Cochin, Service de Parasitologie-Mycologie, Hôpital Cochin, Université de Paris, Paris, France; 12 Roll Back Malaria for West and Central Africa, Kigali, Rwanda; 13 Global Malaria Programme, World Health Organization, Geneva, Switzerland; 14 Division of Infectious Diseases, Department of Medicine, Columbia University Irving Medical Center, New York, NY USA

**Keywords:** Antiparasitic agents, Parasitic infection

Correction to: *Nature Medicine* 10.1038/s41591-020-1005-2, published online 3 August 2020.

In the version of this article initially published, affiliation 2 (Malaria Genetics and Resistance Unit, Institut Pasteur, Paris, France) was incorrect. The correct affiliation is ‘Malaria Genetics and Resistance Unit–Institut Pasteur, INSERM U1201, CNRS ERL9195, Paris, France’. Also, in the first sentence in the first paragraph of the fifth subsection of Results (‘Origins of the Rwandan *Pfkelch13* 561H haplotype and its relationship to other *P. falciparum* populations’), the first part of the sample description (“350 samples, comprising 25 Rwandan sequences and 10 Eritrean *P. falciparum* sequences generated for this study”) was incorrect. The correct text is “...340 samples, comprising 25 Rwandan *P. falciparum* sequences generated for this study...”. Finally, Fig. 1 was incorrect, and the number of worldwide isolates in the legend title (325) was incorrect. The corrected figure is presented here, and the correct number of worldwide isolates is 315. The errors have been corrected in the HTML and PDF versions of the article.

**Fig. 1:**
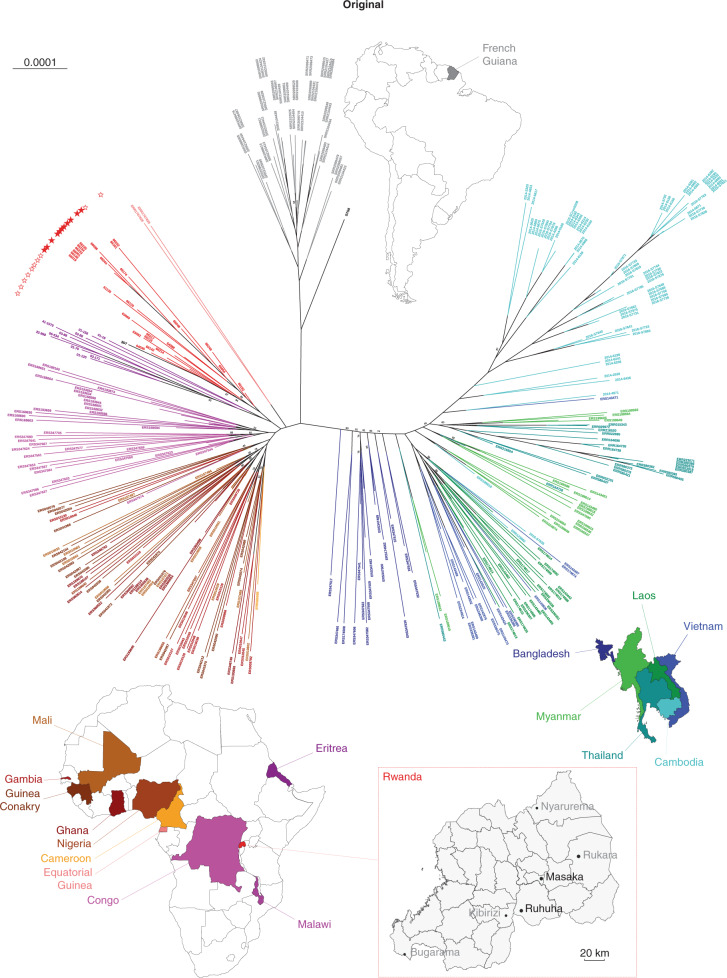

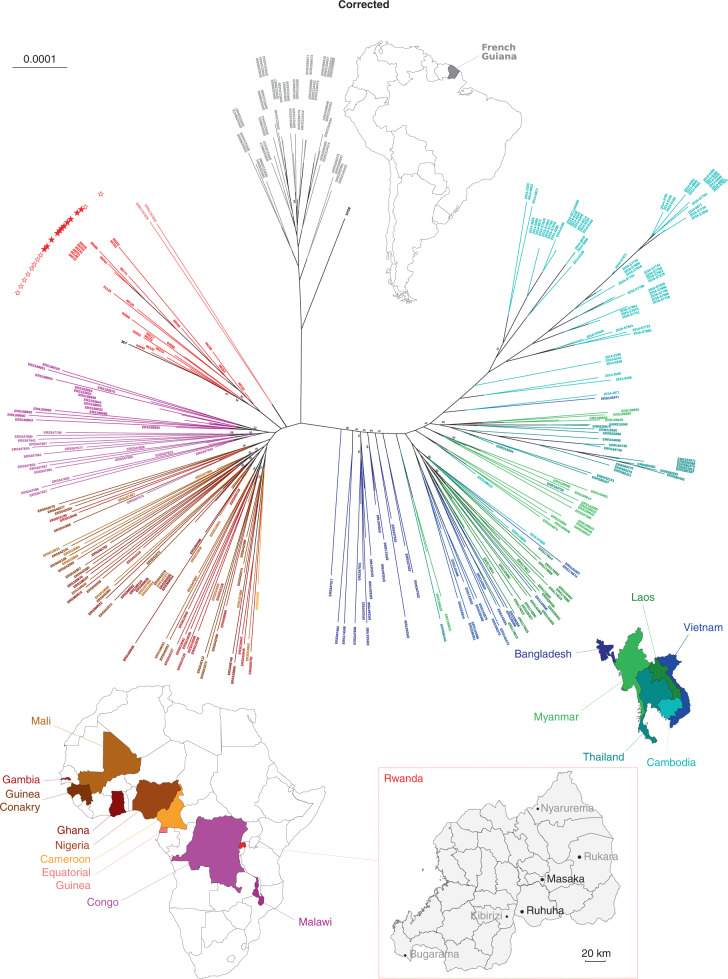
Original and corrected.

