# Supplementary material for: Emergence and clonal expansion of in vitro artemisinin-resistant Plasmodium falciparum kelch13 R561H mutant parasites in Rwanda
Source: Nat Med. 2020 Aug 3;26(10):1602–8. doi: 10.1038/s41591-020-1005-2 (PMC7541349; doi:10.1038/s41591-020-1005-2)
Supplement: Supplementary file 1 — Supplementary Methods 1–7 and Supplementary Tables 1–4. [file 41591_2020_1005_MOESM1_ESM.pdf]

nature medicine

SUPPLEMENTARY INFORMATION

https://doi.org/10.1038/s41591-020-1005-2

In the format provided by the authors and unedited.

Emergence and clonal expansion of in vitro artemisinin-resistant *Plasmodium falciparum kelch13* R561H mutant parasites in Rwanda

In the format provided by the authors and unedited

Emergence and clonal expansion of *in vitro* artemisinin-resistant *Plasmodium falciparum Kelch13* R561H mutant parasites in Rwanda.

Aline Uwimana*, Eric Legrand*, Barbara H. Stokes, Jean-Louis Mangala Ndikumana, Marian Warsame, Noella Umulisa, Daniel Ngamije, Tharcisse Munyaneza, Jean-Baptiste Mazarati, Kaendi Munguti, Pascal Campagne, Alexis Criscuolo, Frédéric Ariey, Monique Murindahabi, Pascal Ringwald, David A. Fidock, Aimable Mbituyumuremyi, Didier Menard

***** Joint first authors.

Technical queries to Didier Menard (dmenard@pasteur.fr)

Supplementary Methods

1. Clinical drug efficacy trial oversight and blood sample collection.
2. *Pfkelch13* genotyping and whole-genome sequencing.
3. Phylogenetic analysis.
4. Genotyping and haplotype analysis.
5. Generation of gene-edited lines.
6. Parasite culture and transfection.
7. Ring-stage survival assay (RSA_{0-3h}).

Supplementary Tables

Table S1. *Pfkelch13* genotyping data on samples collected from patients enrolled in the four additional sites across Rwanda in 2012-2015.
Table S2. Accession identifiers (IDs) of the *P. falciparum* sequences from African locations, Bangladesh, Southeast Asia, or South America (French Guiana), sourced from the MalariaGEN *Plasmodium falciparum* Community Project, https://www.malariagen.net/apps/pf/4.0.
Table S3. Clinical outcomes of the twenty patients harbouring isolates with the *Pfkelch13* R561H allele and molecular markers associated with parasite resistance to piperaquine (*plasmepsin2* gene copy number) or mefloquine (*pfmdr1* gene copy number).
Table S4. Polymorphisms (copy number gene variations and single nucleotide polymorphisms) observed in genes associated with antimalarial drug resistance and in the genetic background common to Southeast Asian artemisinin-resistant parasites. Analysis shown for 24 *P. falciparum* Rwandan isolates (14 *Pfkelch13* 561H mutants and 10 *Pfkelch13* wild-type isolates).

References

Supplementary Methods

1. Clinical drug efficacy trial oversight and blood sample collection.

Clinical studies were coordinated by the Rwanda National Malaria Program and designed to assess the efficacy of artemether-lumefantrine (AL) or dihydroartemisinin-piperaquine (DP) for the treatment of uncomplicated *falciparum* malaria at Masaka and Ruhuha health facilities in 2013-2015 and at Bugarama, Kibirizi, Nyarurema and Rukara health facilities in 2012-2015. The study protocol was approved by the Rwanda National Ethics Committee on 16 May 2012 (RNEC129/RNEC/2012). This study is registered at ISRCTN registry, ISRCTN63145981 (http://www.isrctn.com/ISRCTN63145981), and is now complete.

Briefly, after written informed consent was obtained from the parent/caretaker (or witness' signature and the thumbprint of the participant's parent/caretaker when parent/caretaker was illiterate), children 1-14 years of age presenting with suspected uncomplicated *Plasmodium falciparum* malaria (temperature ≥37.5°C and/or a history of fever within the past 24h) were enrolled in the study if they were subsequently confirmed to have parasitemias ranging from 1,000 to 100,000 parasites per microliter and were able to attend follow-up visits until day 42 post initiation of treatment. Enrolled patients at both sites were randomly assigned to receive a full course of AL (Coartem©, 20 mg artemether and 120 mg lumefantrine per tablet) or DP (Duo-Cotecxin©, 40 mg dihydroarteminisin and 320 mg piperaquine per tablet) according to the manufacturer's dosing schedule.

A blood sample was collected prior to the initiation of treatment (day 0) and was spotted onto filter paper for genotyping. Additional blood samples were collected weekly (on days 7, 14, 21, 28, 35 and 42) during the 42-day follow-up period. Blood samples were also collected in cases of febrile recurrence to differentiate recrudescence (true treatment failure) from new infection. Parasite genotyping was done by polymerase chain reaction (PCR) amplification of *msp1*, *msp2* and *glurp* genes.[1] Thick blood smears were prepared during all follow-up visits and examined to assess parasitemias.

The primary outcome of the study was the PCR-adjusted clinical response to the designated treatment on day 42. Patients were either classified as cured, or in the case of recurrence, as re-infected (new infection) or recrudescent (true treatment failure) according to the WHO 2009 protocol.[2] The secondary outcome was the day 3 positivity rate (day 3+), defined as the proportion of patients who were still parasitemic on day 3 after initiation of treatment as assessed by microscopic examination of thick blood smears.[3]

2. *Pfkelch13* genotyping and whole-genome sequencing.

***Pfkelch13* genotyping.** *Pfkelch13* genotyping aimed at detecting the presence of mutations in the propeller domain of the *Pfkelch13* gene (PF3D7_1343700, codons 440–680, 720 bp), was determined by capillary sequencing of PCR products.[4]

Whole genome sequencing. Capture-based target enrichment of parasite DNA was performed prior to next-generation sequencing (NGS). For each dried blood spot sample, a standard Illumina library was constructed and up to 40 samples were pooled before proceeding to DNA capture. Using SeqCap EZ Prime Developer Probes (Roche Diagnostics

Meylan, France), we developed a pool of biotinylated oligonucleotide probes specific for target regions of interest. The probes were designed based on a concatenation of available *haemosporidia* whole-genome sequences and sequences from organelle genomes (mitochondria and apicoplast) to obtain about 200 Mb of targeted DNA, as presented in the list below. Next, the biotinylated probe/target hybrids were pulled down with streptavidin-coated magnetic beads to obtain libraries that were highly enriched for the targeted regions. Captured DNA molecules were then sequenced with Illumina paired-end sequencing, according to protocols published on the GENOM'IC platform (Institut Cochin, Paris).[5,6] After processing, data were integrated into the Whole-Genome Data Manager (version 2.0).[7] Genomes with a minimum mean depth coverage of 30× and with a minimum of 40% of the genome having at least 10× coverage were retained.

List of available *haemosporidia* whole-genome sequences.

**Reference of complete genomes**	Median total length (Mb)
Laverania	
Plasmodium falciparum	24.62
Plasmodium gaboni	18.34
Plasmodium vivax	28.89
Plasmodium ovale wallikeri	36.03
Plasmodium knowlesi	23.95
Plasmodium malariae	31.92
***Plasmodium* in Rodents**	
Plasmodium vinckei	18.57
Plasmodium yoelii	22.48
***Plasmodium* in Birds**	
Plasmodium gallinaceum strain 8A	25.03
***Haemoproteus* in Birds**	
Haemoproteus tartakovskyi	23.20

3. Phylogenetic analysis.

Each of the 350 read alignments (genome reference sequence: *P. falciparum* 3D7 v45) were used to infer 14 pseudo-chromosomes following four rules: (i) only aligned reads and sequenced bases associated with a Phred score > 20 were considered; (ii) each reference position was replaced by the character state(s) observed in > 80% of the aligned ones at that position, (iii) any reference position with < 6 aligned reads was considered as under-covered and replaced by the unknown character state '?'; and (iv) all polymorphic positions located within a strand-biased position (set as < 3 aligned reads on at least one strand) or close to an under-covered position were replaced by the character state 'X'.

All inferred pseudo-chromosomes were pooled into 14 matrices of aligned nucleotide characters, and every aligned character containing >10% undefined character states (i.e. '?', '-', 'X' or 'N') was discarded. The 14 resulting multiple sequence alignments were concatenated into a supermatrix of 17,313,072 nucleotide characters. Phylogenetic analysis was carried out using IQ-TREE v1.6.7.2 with evolutionary model GTR+FO+R10 and SH-aLRT branch supports (1,000 replicates).[8,9]

4. Genotyping and Haplotype analysis.

The Genome Analysis Toolkit (GATK) Haplotype Caller (version 4.1.7.0) was used to identify single nucleotide polymorphisms (SNPs) in isolates following GATK best practices.[10] Low-quality sites were excluded based primarily on two parameters: GQ ≥ 30 and QUAL ≥ 40. Lastly, calls from sites exhibiting a low call rate across the whole dataset were discarded. Based on genotyped data, the genetic identities of *Pfkelch13* mutants from Rwanda and Asia were assessed by comparing alleles at loci within a 200 kb window around the R561H mutation. A Principal Coordinate Analysis (PCoA) was performed by computing pairwise Euclidean genetic distances among samples in the same 494 kb window. Lastly, the extent to which mutant haplotypes originated from Rwanda was estimated by recording the number of discrepancies between each haplotype and the mutant consensus sequence.

5. Generation of gene-edited lines.

To evaluate the *in vitro* susceptibilities of *Pfkelch13* R561H and *Pfkelch13* P574L mutant parasites to dihydroartemisinin, CRISPR-Cas9 was used to genetically modify the *Pfkelch13* locus in the Dd2 reference line, resulting in the $Dd2^{R561H}$ and $Dd2^{P547L}$ lines. To achieve this, a *Pfkelch13*-propeller domain-specific guide RNA (gRNA) was cloned into an all-in-one CRISPR-Cas9 vector at the BbsI restriction sites using the primer pair p1/p2 (see list below) to generate the pDC2-cam-coSpCas9-U6-gRNA-hdhfr plasmid.[11] *Pfkelch13* donor sequences harbouring the mutations of interest were generated by site-directed mutagenesis of a 1.5 kb region of the *Pfkelch13*-propeller domain, which was amplified using the primer pair p3/p4 and cloned into the pGEM T-easy vector system (Promega). Silent shield mutations were introduced at the Cas9 cleavage site using the primer pair p5/p6, and allele-specific mutations were introduced using the primer pairs p7/p8 for the R561H mutation and p9/p10 for the P574L mutation. *Pfkelch13* donor sequences were amplified from pGEM using the primer pair p11/p12 and sub-cloned into the pDC2-cam-coSpCas9-U6-gRNA-hdhfr at the EcoRI and AatII restriction sites by In-Fusion® Cloning (Takara) to generate the pDC2-cam-coSpCas9-U6-gRNA-*Pfkelch13*^{R561H}-hdhfr and pDC2-cam-coSpCas9-U6-gRNA- *Pfkelch13*^{P574L}-hdhfr plasmids. The final plasmids were sequenced using primers p13, p14 and p15.

List of oligonucleotides used for the generation of gene-edited lines.

Name	Nucleotide sequence (5'-3')	Description	Fidock Lab Name
p1	TATTACACATAGCTGATGATCTAG	*Pfkelch13* gRNA fwd	p6558
p2	AAACCTAGATCATCAGCTATGTGT	*Pfkelch13* gRNA rev	p6287
p3	GTGACGTCGATTGATATTAATGTTG GTGGAGC	*Pfkelch13* CRISPR/Cas9 donor amplification fwd	p3984
p4	CCGCATATGGTGCAAACGGAGTGA CCAAATCTGGG	*Pfkelch13* CRISPR/Cas9 donor amplification rev	p3986
p5	GAATACGCCAAGATCATCAGCTAT GTGTGTTGCTTTTGATAATAAAATT TATGTCATTGG	SDM *Pfkelch13* shield mutations fwd	p6090
p6	GCAACACACATAGCTGATGATCTT GGCGTATTCAAAGGTGCCACCTCTA CCC	SDM *Pfkelch13* shield mutations rev	p6091
p7	GCATATGATCATCATATGAAAGCA TGGGTAGAGGTGGCACCTTTGAAT ACCCC	SDM *Pfkelch13* R561H fwd	p5176

p8	CCCATGCTTTCATATGATGATCATATGCTTCTACATTCGGTATAATAGAAGAGCC	SDM *Pfkelch13* R561H rev	p5176
p9	GAATACGCTAAGATCATCAGCTATGTGTGTTGCTTTTGATAATAAAATTTATGTCATTGG	SDM *Pfkelch13* P574L + shield fwd	p6092
p10	GCAACACACATAGCTGATGATCTTAGCGTATTCAAAGGTGCCACCTCTACCC	SDM *Pfkelch13* P574L + shield rev	p6093
p11	GAGGTACCGAGCTCGAATTCGAAACGGAATTAAGTGATGCTAG	*Pfkelch13* CRISPR/Cas9 donor EcoRI In-Fusion fwd	p6655
p12	CGAAAAGTGCCACCTGACGTCAAACGGAGTGACCAAATCTGGG	*Pfkelch13* CRISPR/Cas9 donor AatII In-Fusion rev	p6656
p13	AACATATGTTAAATATTTATTTCTC	CRISPR/Cas9 donor sequencing fwd	p282
p14	AGGGTTATTGTCTCATGAGCGG	CRISPR/Cas9 donor sequencing fwd	p283
p15	AAGCACCGACTCGGTGCCAC	gRNA sequencing rev	p35
p16	GGGAATCTGGTGGTAACAGC	*Pfkelch13* integration primer fwd (5' end)	p6176
p17	CGGAGTGACCAAATCTGGGA	*Pfkelch13* integration primer rev (3' end)	p6175
p18	GGTATTAAATTTTTACCATTCCCATTAGTATTTTGTATAGG	*Pfkelch13* sequencing fwd (internal)	p4186

fwd, forward; rev, reverse; SDM, site-directed mutagenesis

6. Parasite culture and transfection.

P. falciparum asexual blood-stage parasites were cultured in human erythrocytes (at 3% hematocrit) and RPMI-1640 medium supplemented with 2 mM L-glutamine, 50 mg/L hypoxanthine, 25 mM HEPES, 0.225% $NaHCO_3$, 10 mg/L gentamycin and 0.5% w/v Albumax II (Invitrogen). Parasites were maintained at 37ºC in 5% O2, 5% CO2, and 90% N2. Cultures were monitored by blood smears fixed in methanol, stained with Giemsa, and viewed by light microscopy.

Transfections were performed by electroporating ring-stage parasites at 5–10% parasitemia with 50 µg of purified circular plasmid DNA resuspended in Cytomix. Transfected parasites were maintained under 2.5 nM WR99210 (Jacobus Pharmaceuticals) to select for editing events. Parasite cultures were monitored for recrudescence microscopically for up six weeks post electroporation. To test for successful editing, the *Pfkelch13* locus was amplified directly from whole blood using the primer pair p16/p17 and the MyTaq™ Blood-PCR Kit (Bioline Meridian Bioscience, Cincinnati, USA). PCR products were submitted for Sanger sequencing using primer p18. Bulk-edited cultures were cloned via limiting dilution and flow cytometry was used to screen for positive wells after 17-20 days. Parasites were stained with 1× SYBR Green (Thermo Fisher, Waltham, USA) and 100 nM MitoTracker Deep Red (Invitrogen, Grand Island, USA), and positive wells were detected by flow cytometry using an iQue flow cytometer (IntelliCyt, Albuquerque, USA).

7. Ring-stage survival assays ($RSA_{0\text{-}3h}$).

In vitro ring-stage survival assays ($RSA_{0\text{-}3h}$) were conducted on very early ring-stage parasites (0-3 hours post-invasion; hpi).[12] These assays included the $Dd2^{R561H}$ and $Dd2^{P574L}$ CRISPR/Cas9-edited lines, as well as the $Dd2^{WT}$ and the $Dd2^{C580Y}$ lines as controls.[13] Briefly, tightly synchronized 0-3 hpi rings were exposed to a pharmacologically-relevant dose of 700 nM dihydroartemisinin (DHA) or 0.1% dimethyl sulfoxide (DMSO; vehicle control) for 6h, washed three times with RPMI to remove drug, transferred, and cultured for an addition 66h in drug-free medium. Media was removed parasite culture resuspended on a Freedom Evo 100 liquid-handling instrument (Tecan). Parasitemias were measured at 72h by flow cytometry with 50,000-100,000 events captured per sample. Parasite survival rates are expressed as ratios of viable parasites in DHA- to DMSO-treated samples. The threshold for resistance in the RSA was considered to be survival rates >1%.

Supplementary Tables

Table S1. *Pfkelch13* genotyping data on samples collected from patients enrolled in the four additional sites across Rwanda in 2012-2015.

Samples	Bugarama	Kibirizi	Nyarurema	Rukara
Samples size	112	101	73	134
Pfkelch13 genotyping				
WT	110	101	69	125
Synonymous mutations*	1	0	1	3
469 (C→F)			1	
487 (V→I)			1	
555 (V→A)				4
**561 (R→H)**				**1**
578 (A→S)			1	
578 (A→V)	1			
667 (P→R)				1

* Synonymous mutations were C469C (N=1, detected in Rukara), S477S (N=1, detected in Rukara), V510V (N=1, detected in Bugarama) and V666V (N=2, detected in Nyarurema and Rukara). The R561H non-synonymous mutation, shown in bold font, has been previously associated with delayed parasite clearance in Southeast Asia.[3]

Table S2. Accession identifiers (IDs) of the *P. falciparum* sequences from African locations, Bangladesh, Southeast Asia, or South America (French Guiana), sourced from the MalariaGEN *Plasmodium falciparum* Community Project; https://www.malariagen.net/apps/pf/4.0

Location	Sample Accession ID
Reference lines	3D7, 7G8
Bangladesh	ERS174508, ERS174562, ERS347460, ERS347469, ERS347492, ERS347500, ERS347508, ERS347509, ERS347515, ERS347517, ERS347532, ERS347533, ERS347541, ERS347582, ERS347606, ERS347622, ERS347630
Cambodia	2014-2320_S1, 2014-2539_S4, 2014-3625_S6, 2014-3670_S7,2014-3792_S8, 2014-4281_S9, 2014-4288_S10, 2014-4392_S11, 2014-4653_S13, 2014-

	4971_S14, 2014-4992_S15, 2014-5168_S17, 2014-5817_S18, 2014-5865_S20, 2014-5968_S21, 2014-6139_S22, 2014-6150_S23, 2014-6158_S24, 2014-6160_S26, 2014-6168_S27, 2014-6199_S28, 2014-6208_S29, 2014-6214_S30, 2014-6230_S31, 2014-6241_S32, 2014-6282_S33, 2014-6290_S34, 2014-6306_S35, 2014-6332_S36, 2014-6343_S37, 2014-6436_S39, 2014-6870_S40, 2014-6871_S41, 2014-6889_S42, 2014-6891_S43, 2016-S7629, 2016-S7640, 2016-S7641, 2016-S7649, 2016-S7661, 2016-S7686, 2016-S7695, 2016-S7701, 2016-S7708, 2016-S7723, 2016-S7728, 2016-S7730, 2016-S7731, 2016-S7733, 2016-S7744, 2016-S7763, 2016-S7790, 2016-S7794, 2016-S7795, 2016-S7809, 2016-S7818, 2016-S7821, 2016-S7822, 2016-S7835, 2016-S7836, 2016-S7850, 2016-S7871, 2016-S7875, 2016-S7876, 2016-S7878, 2016-S7883, 2016-SCryo5208, ERR123882
Cameroon	ERR580585
DR Congo	ERS347544, ERS347560, ERS347561, ERS347567, ERS347577, ERS347584, ERS347596, ERS347603, ERS347623, ERS347624, ERS347637, ERS347641, ERS347653, ERS347659, ERS347674, ERS347680, ERS347687, ERS347705
French Guiana	SRR2098005, SRR2098351, SRR2098613, SRR2098911, SRR2098915, SRR2098950, SRR2098951,SRR2099471, SRR2099473, SRR2099476, SRR2099479, SRR2099480, SRR2099481,SRR2099529, SRR2099549, SRR2099550, SRR2099557, SRR2099563, SRR2099776,SRR2099884, SRR2099889, SRR2101832, SRR2102025, SRR2103413, SRR2103599,SRR2103608, SRR2103639, SRR2104404, SRR2104409, SRR2104410, SRR2104411,SRR2104414, SRR2104415, SRR2104423, SRR2104424, SRR2104427, SRR2104428,SRR2104431, SRR2104433, SRR2104434, SRR2104438, SRR2104439, SRR2106474,SRR2106487, SRR2106507
The Gambia	ERS009726, ERS009782, ERS009966, ERS010038, ERS010039, ERS010040, ERS010043, ERS010045, ERS010047, ERS010048, ERS010049, ERS010119, ERS010126, ERS010127, ERS010128, ERS010130
Ghana	ERS188107, ERS188114, ERS193645, ERS193655, ERS193670, ERS346749, ERS346792, ERS360498, ERS360514, ERS360522, ERS360530, ERS360538, ERS360554, ERS360562
Guinea Conakry	ERS041968, ERS041970, ERS041972, ERS041974, ERS041976, ERS042038, ERS042050, ERS042054, ERS042057, ERS042074, ERS042077, ERS042079, ERS042083, ERS042085, ERS042089, ERS042090, ERS042097, ERS042104, ERS042108, ERS042112
Guinea Equatorial	ERR1767828, ERR1767829
Laos	ERS143442, ERS143455, ERS143460, ERS143482, ERS174514, ERS174515, ERS174585, ERS174588, ERS174592, ERS174599, ERS174602, ERS174613, ERS174614, ERS174615, ERS174617, ERS174625, ERS336338, ERS336351, ERS336357
Malawi	ERS168594, ERS168596, ERS168599, ERS168600, ERS168601, ERS168603, ERS168604, ERS168630, ERS168632, ERS168651, ERS188100, ERS193634, ERS193644, ERS193654, ERS193659, ERS193674
Mali	ERS010024, ERS010029, ERS010031, ERS010033, ERS010563, ERS010652, ERS010658, ERS010663, ERS010666, ERS157471, ERS157489, ERS157493, ERS157494, ERS157497
Myanmar	ERR246546, ERR246547, ERS143451, ERS143480, ERS199593, ERS199594, ERS199598, ERS199605, ERS199609, ERS199614, ERS199615, ERS199618, ERS199619, ERS199623, ERS199633, ERS199642, ERS199649, ERS224874, ERS224884, ERS347690
Nigeria	ERS199640, ERS199650, ERS347697
Thailand	ERR015343, ERR029972, ERR164698, ERR164735, ERR164738, ERR216520, ERR223071, ERR553918, ERR580375, ERR580376, ERR580379, ERR580382, ERR580387, ERR580389, ERR580395, ERR580405, ERR580421, ERR580443
Vietnam	ERS142875, ERS143424, ERS143469, ERS143471, ERS143473, ERS143497, ERS143499, ERS143502, ERS143506, ERS143520, ERS174674, ERS224924, ERS336368, ERS336375, ERS336380, ERS336381, ERS336386

Table S3. Clinical outcomes of the twenty patients harbouring isolates with the *Pfkelch13* R561H allele and molecular markers associated with parasite resistance to piperaquine (*plasmepsin2* gene copy number) or mefloquine/lumefantrine (*pfmdr1* gene copy number).

Patient ID	Sampling site	Year of collection	Treatment arm	Day 0 parasitemia (parasite per µL of blood)	Day 3 parasitemia (parasite per µL of blood)	Clinical outcome at Day 42	*Plasmepsin 2* gene copy number	*mdr1* gene copy number
66019	Masaka	2014	AL	30560	0	Reinfection*	NA	NA
66032	Masaka	2014	AL	1200	0	Reinfection*	NA	NA
66033	Masaka	2014	AL	10000	0	Reinfection*	NA	NA
66044	Masaka	2014	DP	2800	0	ACPR	single copy	single copy
66048	Masaka	2014	DP	1920	0	ACPR	single copy	single copy
66051	Masaka	2014	DP	7400	0	ACPR	single copy	single copy
66054	Masaka	2014	DP	27200	0	ACPR	single copy	single copy
66086	Masaka	2015	DP	32000	0	ACPR	single copy	single copy
66120	Masaka	2015	AL	1600	0	ACPR	NA	NA
66148	Masaka	2015	AL	36000	0	ACPR	NA	NA
66149	Masaka	2015	DP	8000	0	ACPR	NA	NA
66161	Masaka	2015	DP	54000	200	Reinfection*	single copy	single copy
66174	Masaka	2015	AL	26000	0	Reinfection*	NA	NA
66202	Masaka	2015	AL	1000	0	Reinfection*	NA	NA
66233	Masaka	2015	DP	4800	0	Reinfection*	single copy	single copy
66234	Masaka	2015	AL	1200	0	ACPR	NA	NA
66235	Masaka	2015	AL	28000	0	Reinfection*	NA	NA
66249	Masaka	2015	DP	20000	0	ACPR	single copy	single copy
66254	Masaka	2015	DP	1000	0	Reinfection*	single copy	single copy
61135	Rukara	2015	AL	20480	0	Recrudescence at Day 21	NA	NA

* Patients classified as re-infected after PCR genotyping were excluded from the final per protocol analysis. AL, artemether + lumefantrine; DP, dihydroartemisinin + piperaquine; ACPR (Adequate Clinical and Parasitological Response): Patients classified as cured at day 42. NA: Not available

Table S4. Polymorphisms (copy number gene variations and single nucleotide polymorphisms) observed in genes associated with antimalarial drug resistance and in the genetic background common to Southeast Asian artemisinin-resistant parasites. Analysis shown for 24 *P. falciparum* Rwandan isolates (14 *Pfkelch13* 561H mutants and ten *Pfkelch13* wild-type isolates).

**Genomic change**	**Gene**	**Gene ID**	**Polymorphism**	**561H**	**WT**	***p*-value**
CNV	*pfmdr1*	PF3D7_0523000	≥2	0/14	0/10	0.44*
	plasmepsin2	PF3D7_1408000	≥2	0/14	0/10	0.6*
SNP	*pfcrt*	PF3D7_0709000	wild-type	14/14	6/10	0.08**
			74I/75E/76T/271E	0/14	1/10	
			74I/75E/76T/220S/271E/371I	0/14	2/10	
			74I/75E/76T/141L/220S/271E/371I	0/14	1/10	
	pfmdr1	PF3D7_0523000	wild-type	2/14	5/10	0.14**
			184Y	11/14	5/10	
			184Y/1246Y	1/14	0/10	
	dhfr	F3D7_0417200	51I/108N	1/14	2/10	0.08**
			51I/59R/108N	12/14	5/10	
			51I/59R/108N/164L	1/14	3/10	
	dhps	PF3D7_0810800	437A	0/14	1/9	0.22**
			540E	1/14	2/9	
			540E/581G	13/14	6/9	
	cytochrome b	mal_mito_3	wild-type	14/14	10/10	0.41*
	mrp-1	PF3D7_0112200	wild-type	1/14	3/10	0.05**
			876V	1/14	0/10	
			1466R	3/14	5/10	
			876V/1466R	9/14	1/10	
			1172E/1364V	0/14	1/10	
Genetic background	*ferredoxin*	PF3D7_1318100	D193Y	2/13	2/9	0.69*
	apicoplast ribosomal protein S10 precursor	PF3D7_1460900	V127M	0/14	0/9	0.3*
	multidrug resistance protein 2	PF3D7_1447900	T484I	0/13	0/10	0.53*
	pib7	PF3D7_0720700	C1484F	0/14	0/9	0.29*
	pph	PF3D7_1012700	V1157L	0/14	0/9	0.29*
	exonuclease, putative	PF3D7_1362500	E415G	0/14	0/9	0.29*

CNV: Copy number variation; SNP: Single nucleotide polymorphism.
* Fisher's exact test (one-sided); ** Chi-squared test (one-sided)

References

1. Snounou, G. & Beck, H.P. The use of PCR genotyping in the assessment of recrudescence or reinfection after antimalarial drug treatment. *Parasitol. Today* **14**, 462-7 (1998).

2. World Health Organization. Methods and techniques for clinical trials on antimalarial drug efficacy: Genotyping to identify parasite populations. Geneva, Switzerland. https://www.who.int/malaria/publications/atoz/9789241596305/en (2018).

3. World Health Organization. Status report on artemisinin resistance and ACT efficacy Geneva, Switzerland. https://www.who.int/malaria/publications/atoz/artemisinin-resistance-august2018/en (2019).

4. Menard, D. *et al.* A worldwide map of *Plasmodium falciparum* K13-propeller polymorphisms. *N. Engl. J. Med.* **374**, 2453-64 (2016).

5. Ariey, F. *et al.* A molecular marker of artemisinin-resistant *Plasmodium falciparum* malaria. *Nature* **505**, 50-5 (2014).

6. Witkowski, B. *et al.* A surrogate marker of piperaquine-resistant *Plasmodium falciparum* malaria: a phenotype-genotype association study. *Lancet Infect. Dis.* **17**, 174-83 (2017).

7. Beghain, J. *et al. Plasmodium* copy number variation scan: gene copy numbers evaluation in haploid genomes. *Malar. J.* **15**, 206 (2016).

8. Guindon, S. *et al.*. New algorithms and methods to estimate maximum-likelihood phylogenies: assessing the performance of PhyML 3.0. *Syst. Biol.* **59**, 307-21 (2010).

9. Nguyen, L.T., Schmidt, H.A., von Haeseler, A. & Minh, B.Q. IQ-TREE: a fast and effective stochastic algorithm for estimating maximum-likelihood phylogenies. *Mol. Biol. Evol.* **32**, 268-74 (2015).

10. Van der Auwera, G.A. *et al.* From FastQ data to high confidence variant calls: the Genome Analysis Toolkit best practices pipeline. *Curr. Protoc. Bioinformatics* **43**, 11.10.1-11.10.33 (2013).

11. Ng, C.L. & Fidock, D.A. *Plasmodium falciparum in vitro* drug resistance selections and gene editing. *Methods Mol. Biol.* **2013**, 123-40 (2019).

12. Witkowski, B. *et al.* Novel phenotypic assays for the detection of artemisinin-resistant *Plasmodium falciparum* malaria in Cambodia: *in-vitro* and *ex-vivo* drug-response studies. *Lancet Infect. Dis.* **13**, 1043-9 (2013).

13. Straimer, J. *et al.* K13-propeller mutations confer artemisinin resistance in *Plasmodium falciparum* clinical isolates. *Science* **347**, 428-31 (2015).
