# Supplementary material for: Emergence and clonal expansion of in vitro artemisinin-resistant Plasmodium falciparum kelch13 R561H mutant parasites in Rwanda
Source: Nat Med. 2020 Aug 3;26(10):1602–8. doi: 10.1038/s41591-020-1005-2 (PMC7541349; doi:10.1038/s41591-020-1005-2)
Supplement: Supplementary file 2 — Reporting Summary [file 41591_2020_1005_MOESM2_ESM.pdf]

Corresponding author(s): Didier MENARD

Last updated by author(s): Jun 24, 2020

Reporting Summary

Nature Research wishes to improve the reproducibility of the work that we publish. This form provides structure for consistency and transparency in reporting. For further information on Nature Research policies, see Authors & Referees and the Editorial Policy Checklist.

Statistics

For all statistical analyses, confirm that the following items are present in the figure legend, table legend, main text, or Methods section.

n/a	Confirmed	
☐	☒	The exact sample size (*n*) for each experimental group/condition, given as a discrete number and unit of measurement
☐	☒	A statement on whether measurements were taken from distinct samples or whether the same sample was measured repeatedly
☐	☒	The statistical test(s) used AND whether they are one- or two-sided<br>*Only common tests should be described solely by name; describe more complex techniques in the Methods section.*
☒	☐	A description of all covariates tested
☒	☐	A description of any assumptions or corrections, such as tests of normality and adjustment for multiple comparisons
☐	☒	A full description of the statistical parameters including central tendency (e.g. means) or other basic estimates (e.g. regression coefficient) AND variation (e.g. standard deviation) or associated estimates of uncertainty (e.g. confidence intervals)
☐	☒	For null hypothesis testing, the test statistic (e.g. *F*, *t*, *r*) with confidence intervals, effect sizes, degrees of freedom and *P* value noted<br>*Give P values as exact values whenever suitable.*
☒	☐	For Bayesian analysis, information on the choice of priors and Markov chain Monte Carlo settings
☒	☐	For hierarchical and complex designs, identification of the appropriate level for tests and full reporting of outcomes
☒	☐	Estimates of effect sizes (e.g. Cohen's *d*, Pearson's *r*), indicating how they were calculated

Our web collection on statistics for biologists contains articles on many of the points above.

Software and code

Policy information about availability of computer code

Data collection	Microsoft Excel (Office 2016)
Data analysis	MedCal (version 12), Prism (version 8), Whole-genome Data Manager (version 2.0), IQ-TREE v1.6.7.2 with evolutionary model GTR+FO +R10 and SH-aLRT branch supports (1,000 replicates), Genome Analysis Toolkit (GATK) Haplotype Caller (4.1.7.0).

For manuscripts utilizing custom algorithms or software that are central to the research but not yet described in published literature, software must be made available to editors/reviewers. We strongly encourage code deposition in a community repository (e.g. GitHub). See the Nature Research guidelines for submitting code & software for further information.

Data

Policy information about availability of data

All manuscripts must include a data availability statement. This statement should provide the following information, where applicable:

- Accession codes, unique identifiers, or web links for publicly available datasets
- A list of figures that have associated raw data
- A description of any restrictions on data availability

The data that support the findings of this study are available from the corresponding authors upon reasonable request. Parasite whole-genome sequences have been deposited in repository https://www.ncbi.nlm.nih.gov/bioproject/PRJEB38946 and the sequence files are accessible under the accession numbers ERS4758427 – ERS4758451.

Field-specific reporting

Please select the one below that is the best fit for your research. If you are not sure, read the appropriate sections before making your selection.

☒ Life sciences ☐ Behavioural & social sciences ☐ Ecological, evolutionary & environmental sciences

For a reference copy of the document with all sections, see nature.com/documents/nr-reporting-summary-flat.pdf

Life sciences study design

All studies must disclose on these points even when the disclosure is negative.

Sample size	Sample size calculations were informed by results from the 2009 study on AL and DHP conducted in Rwanda (The Four Artemisinin-Based Combinations (4ABC) Study Group. A head-to-head comparison of four artemisinin-based combinations for treating uncomplicated malaria in African children: a randomized trial. PLoS Med 2011;8(11):e1001119). Using a two-sided type I error rate of 0.05 and an 80% power to detect a 5% difference between treatments, a sample of 268 patients per treatment arm was used. The total sample for each treatment arm was split evenly between the two study sites.
Data exclusions	In the clinical data analysis, a per-protocol analysis was conducted excluding patients with new infections during the follow-up period to calculate the proportion of the ACPR in the PCR-adjusted data set. Data were excluded from the PCR-adjusted analyses if the genotyping results were unclassifiable or identified a new infection. The exclusion criteria were pre-established.
Replication	All attempts at replication were successful (see data, Figure 2)
Randomization	A randomization list was computer generated for different age-strata (<2 years; 2-5 years; 5-10; 10-14 years) using MS-Excel. Sequentially numbered sealed envelopes containing the treatment group assignments were prepared from the randomization list for each age category. The study doctor assigned a study number to the participant and the study nurse administered treatment by opening the envelope corresponding to the treatment number.
Blinding	The randomization codes were secured in a locked cabinet accessible only by the study nurse. Only the study nurse and patients were aware of treatment assignments whereas the study doctor was blinded to the treatment assignments

Reporting for specific materials, systems and methods

We require information from authors about some types of materials, experimental systems and methods used in many studies. Here, indicate whether each material, system or method listed is relevant to your study. If you are not sure if a list item applies to your research, read the appropriate section before selecting a response.

Materials & experimental systems

n/a	Involved in the study	
☒	☐	Antibodies
☐	☒	Eukaryotic cell lines
☒	☐	Palaeontology
☒	☐	Animals and other organisms
☐	☒	Human research participants
☐	☒	Clinical data

Methods

n/a	Involved in the study	
☒	☐	ChIP-seq
☒	☐	Flow cytometry
☒	☐	MRI-based neuroimaging

Eukaryotic cell lines

Policy information about cell lines

Cell line source(s)	Plasmodium falciparum Dd2 line - MRA 156 - https://www.beiresources.org/Catalog/BEIParasiticProtozoa/MRA-156.aspx
Authentication	The authentication procedure of P. falciparum Dd2 line is described in the certificate of analysis for MRA-156 - https://www.beiresources.org/Catalog/BEIParasiticProtozoa/MRA-156.aspx#
Mycoplasma contamination	P. falciparum Dd2 cell line was tested negative for mycoplasma contamination
Commonly misidentified lines (See ICLAC register)	No misidentified lines

Human research participants

Policy information about studies involving human research participants

Population characteristics	Children 1-14 years of age presenting with suspected uncomplicated Plasmodium falciparum malaria (temperature ≥37.5°C and/or a history of fever within the past 24h).
Recruitment	Children 1-14 years of age were enrolled if they were subsequently confirmed to have parasitemias ranging from 1,000 to 100,000 parasites per microliter and were able to attend follow-up visits until day 42 post initiation of treatment. Enrolled patients were randomly assigned to receive a full course of AL (Coartem©, 20 mg artemether and 120 mg lumefantrine per tablet) or DP (Duo-Cotecxin©, 40 mg dihydroarteminisin and 320 mg piperaquine per tablet) according to the manufacturer's dosing schedule. A blood sample was collected prior to the initiation of treatment (day 0) and was spotted onto filter paper for genotyping. Additional blood samples were collected weekly (on days 7, 14, 21, 28, 35 and 42) during the 42-day follow-up period. Blood samples were also collected in cases of febrile recurrence to differentiate recrudescence (true treatment failure) from new infection.
Ethics oversight	We confirm that this clinical study was performed in accordance with relevant guidelines and regulations. Approval for conducting the study was obtained from the Rwandan National Ethics Committee (RNEC129/RNEC/2012).

Note that full information on the approval of the study protocol must also be provided in the manuscript.

Clinical data

Policy information about clinical studies

All manuscripts should comply with the ICMJE guidelines for publication of clinical research and a completed CONSORT checklist must be included with all submissions.

Clinical trial registration	ISRCTN63145981 (http://www.isrctn.com/ISRCTN63145981)
Study protocol	The study protocol was approved by the Rwanda National Ethics Committee on 16 May 2012 (RNEC129/RNEC/2012). The full trial protocol is available from the corresponding authors upon request.
Data collection	Data were collected from clinical studies, coordinated by the Rwanda National Malaria Program and designed to assess the efficacy of artemether-lumefantrine (AL) or dihydroartemisinin-piperaquine (DP) for the treatment of uncomplicated falciparum malaria at Masaka and Ruhuha health facilities in 2013-2015 and at Bugarama, Kibirizi, Nyarurema and Rukara health facilities in 2012-2015.
Outcomes	The primary and secondary outcomes were pre-defined. The primary outcome of the study was the PCR-adjusted clinical response to the designated treatment on day 42. Patients were either classified as cured, or in the case of recurrence, as re-infected (new infection) or recrudescent (true treatment failure) according to the WHO 2009 protocol. The secondary outcome was the day 3 positivity rate (day 3+), defined as the proportion of patients who were still parasitemic on day 3 after initiation of treatment as assessed by microscopic examination of thick blood smears.
